# Pharmacological Activation of p53 during Human Monocyte to Macrophage Differentiation Attenuates Their Pro-Inflammatory Activation by TLR4, TLR7 and TLR8 Agonists

**DOI:** 10.3390/cancers13050958

**Published:** 2021-02-25

**Authors:** Dmitry Namgaladze, Bernhard Brüne

**Affiliations:** 1 Institute of Biochemistry I, Faculty of Medicine, Goethe-University Frankfurt, 60590 Frankfurt, Germany; B.Bruene@biochem.uni-frankfurt.de; 2 Fraunhofer Institute for Translational Medicine and Pharmacology ITMP, 60590 Frankfurt, Germany; 3 German Cancer Consortium (DKTK), Partner Site Frankfurt, 60590 Frankfurt, Germany; 4 Frankfurt Cancer Institute, Goethe-University Frankfurt, 60590 Frankfurt, Germany

**Keywords:** p53, macrophages, apoptosis, inflammation

## Abstract

**Simple Summary:**

Pharmacological activation of tumor suppressor p53 is a promising therapeutic strategy for a range of hematologic and solid cancers. Whether p53 activation augments or suppresses anti-tumor innate immunity is less understood. Here we show that treatment of differentiating human macrophages with a p53 activator idasanutlin suppresses their inflammatory responses to activators of toll-like receptors (TLR) -4 and -7/8. This is accompanied by reduced expression of TLR7, TLR8, as well as TLR4 co-receptor CD14. These data help evaluating the possibilities of combining p53-targeting and immunostimulatory anti-cancer therapies.

**Abstract:**

The transcription factor p53 has well-recognized roles in regulating cell cycle, DNA damage repair, cell death, and metabolism. It is an important tumor suppressor and pharmacological activation of p53 by interrupting its interaction with the ubiquitin E3 ligase mouse double minute 2 homolog (MDM2) is actively explored for anti-tumor therapies. In immune cells, p53 modulates inflammatory responses, but the impact of p53 on macrophages remains incompletely understood. In this study, we used the MDM2 antagonist idasanutlin (RG7388) to investigate the responses of primary human macrophages to pharmacological p53 activation. Idasanutlin induced a robust p53-dependent transcriptional signature in macrophages, including several pro-apoptotic genes. However, idasanutlin did not generally sensitize macrophages to apoptosis, except for an enhanced response to a Fas-stimulating antibody. In fully differentiated macrophages, idasanutlin did not affect pro-inflammatory gene expression induced by toll-like receptor 4 (TLR4), TLR3, and TLR7/8 agonists, but inhibited interleukin-4-induced macrophage polarization. However, when present during monocyte to macrophage differentiation, idasanutlin attenuated inflammatory responses towards activation of TLR4 and TLR7/8 by low doses of lipopolysaccharide or resiquimod (R848). This was accompanied by a reduced expression of CD14, TLR7, and TLR8 in macrophages differentiated in the presence of idasanutlin. Our data suggest anti-inflammatory effects of pharmacological p53 activation in differentiating human macrophages.

## 1. Introduction

The transcription factor p53 is a tumor suppressor, which is mutated in about 50% of human cancers. Aside from its tumor suppressor function, p53 has important roles in cells of the innate and adaptive immune systems [1,2]. These include p53-dependent regulation of anti-tumor immunity, anti-viral responses, and inflammation. The impact of p53 on inflammatory responses is extensively studied. However, there is still no consensus on whether p53 activity suppresses or supports inflammation. Whereas studies using p53-deficient murine macrophages showed increased inflammatory responses [3,4], treatment of human macrophages with the pharmacological p53 activator Nutlin 3 induced pro-inflammatory chemokine and cytokine expression by activating nuclear factor kappa-light-chain-enhancer of activated B cells (NFκB) [5]. The reasons for this discrepancy are not yet understood. Recently, p53 has also been shown to promote differentiation of CD103^+^ dendritic cells from monocytic progenitors [6]. The impact of p53 on monocyte to macrophage differentiation, especially in the human system, is not understood.

p53 is continuously ubiquitinated by the E3 ubiquitin ligase mouse double minute 2 homolog (MDM2), followed by subsequent proteasomal degradation [7]. Thus, disrupting p53-MDM2 interaction can be pharmacologically exploited to promote p53 stabilization. Nutlin 3 represented the first drug targeting p53-MDM2 interaction [8]. Recently, several antagonists of MDM2-p53 interaction with increased potency and bioavailability compared to Nutlin 3 have been developed [9,10], with some of them advancing to clinical trials. Idasanutlin (RG7388), a potent pyrrolidine-derived MDM2 antagonist developed by Roche [11], is being tested in combinations with chemotherapeutic drugs or Bcl-2 antagonists for the treatment of acute myeloid leukemia (NCT04029688, NCT03850535) [12,13]. How p53 induction by idasanutlin affects the phenotype of human macrophages is not yet described.

In this study, we characterize the influence of idasanutlin on differentiation and inflammatory responses of human monocytes/macrophages. Idasanutlin potently activated p53 in human macrophages, but caused only limited stimulus-specific changes in macrophage viability. Idasanutlin also did not affect inflammatory responses of mature macrophages, while inhibiting transcriptional responses to the Th2 cytokine interleukin (IL)-4. However, when present during monocyte to macrophage differentiation, idasanutlin suppressed inflammatory responses to low doses of lipopolysaccharide (LPS) or the toll-like receptor (TLR) 7/8 agonist R848 (resiquimod). This was accompanied by reduced expression of TLR7/8 as well as the lipopolysaccharide receptor CD14. Our results thus suggest that pharmacological p53 activation in differentiating macrophages may attenuate their ability to respond to pro-inflammatory stimulation.

## 2. Materials and Methods

### 2.1. Monocyte Isolation, Differentiation, and Treatment

Human peripheral blood mononuclear cells (PBMC) were isolated from commercially available buffy coats from anonymous donors (DRK-Blutspendedienst Baden-Württemberg-Hessen, Institut für Transfusionsmedizin und Immunhämatologie, Frankfurt, Germany) using Ficoll (Biochrom, Berlin, Germany) density centrifugation. Monocytes were isolated from PBMC using positive selection with CD14 antibody-coupled magnetic beads (MACS Miltenyi Biotec, Bergisch Gladbach, Germany) following the manufacturer’s protocol. The purity of monocyte preparations was checked by staining with APC-H7-labelled anti-CD14 antibody and flow cytometry, and was routinely higher than 85%. CD14^+^ monocytes were differentiated in macrophage serum-free medium (ThermoFisher Scientific, Waltham, MA, USA) supplemented with 100 U/mL penicillin, 100 μg/mL streptomycin, and 50 ng/mL macrophage colony-stimulating factor (M-CSF) or 50 ng/mL granulocyte-macrophage colony-stimulating factor (GM-CSF) (Immunotools, Friesoythe, Germany) for 7 days. When indicated, cells were treated with RG7388 (Cayman Chemical, Ann Arbor, MI, USA), 1 µg/mL Ultra-LEAF^TM^ Fas-stimulating antibody (305705, Biolegend, San Diego, CA, USA), 10 µg/mL cycloheximide (Sigma-Aldrich, Schnelldorf, Germany), 10 ng/mL tumor necrosis factor α (Peprotech, Hamburg, Germany), 0.1 or 100 ng/mL lipopolysaccharide (LPS, Sigma-Aldrich), 20 ng/mL interleulkin-4 (Peprotech), 25 µg/mL poly(I:C), and 1 or 10 µg/mL R848 (both Invivogen, San Diego, CA, USA).

### 2.2. RNA Isolation and Q-PCR

Total RNA was isolated using TRIzol reagent (Life Technologies, Carlsbad, CA, USA) followed by reverse transcription using Maxima first-strand cDNA synthesis kit (ThermoFisher Scientific). Quantitative real-time polymerase chain reaction (Q-PCR) assays were performed with PowerUp SYBR Green Master Mix (Applied Biosystems, Foster City, CA, USA) using Quant Studio Real Time PCR System (Applied Biosystems). Relative transcript amounts were quantified using Δc_t_ method with β-microglobulin (βMG) as a housekeeping gene (expression = 2^−(Ct(target)−Ct(βMG))^) and normalized to the untreated or cytokine-treated controls. Primer sequences are presented in Table 1.

### 2.3. Western Blot Analysis

Cells were harvested in lysis buffer (50 mM Tris-HCl, pH 8, 150 mM NaCl, 5 mM EDTA, 10 mM NaF, 1 mM Na_3_VO_4_, 0.5% NP-40, 1 mM phenylmethylsulfonyl fluoride (PMSF), protease inhibitor cocktail). Protein lysates were sonicated and centrifuged at 12000 g for 10 min at 4 °C. Supernatants were heat-denatured at 95 °C, separated on SDS-PAGE gels, and transferred onto nitrocellulose membranes. Primary antibodies directed against p53 (#9282), caspase-7 (#9492), phospho-IκB (#2859), IκB (#4814), phospho-JNK (#4668), JNK (#9252) (all Cell Signaling Technology, Frankfurt, Germany), CDKN1A (#sc-397) and nucleolin (#sc-55486, both Santa Cruz Biotechnology, Heidelberg, Germany) were used, followed by IRDye 680 or IRDye 800-coupled secondary antibodies (LICOR Biosciences, Bad Homburg, Germany). Blots were visualized using LICOR ODYSSEY scanner, and analyzed by Image Studio Lite software (LICOR Biosciences). Data used to generate bar diagrams from Western blots and pictures of all Western blots are available for review upon request. Original uncropped Western blot images used in the figures are provided as Figure S1.

### 2.4. Caspase Activity Assay

Cells were harvested in caspase activity buffer (100 mM HEPES, pH 7.5, 10% sucrose, 0.1% CHAPS, 1 mM EDTA), sonicated, and centrifuged at 13000 g for 10 min at 4 °C; 30 µg protein from the supernatants were then diluted with caspase activity buffer containing 1 mM DTT, and incubated in a 96-well plate with 100 µM caspase substrate N-Acetyl-Asp-Glu-Val-Asp-7-amido-4-methylcoumarin (Ac-DEVD-AMC, Sigma-Aldrich). Fluorescence was then continuously recorded at 30 °C using a Tecan Spark (Tecan, Männedorf, Switzerland) microplate reader.

### 2.5. Flow Cytometry

Macrophages were harvested, washed with PBS, and pelleted at 500 g at 4 °C for 5 min. Cells were blocked with 2% FcR blocking reagent (Miltenyi Biotec) in PBS for 10 min followed by stainings with APC-H7-labelled anti-CD14 antibody (560180, BD Biosciences, Heidelberg, Germany) for 20 min on ice in the dark. Samples were washed and analyzed using a FACSymphony A5 flow cytometer (BD Biosciences).

### 2.6. Statistical Analysis

Statistical analysis was performed using GraphPad (San Diego, CA, USA) Prism 8.0. Data were analyzed using student paired, two-tailed t test or by one-way ANOVA with Bonferoni multiple comparisons. Graphical data are presented as means ± SEM for at least three independent experiments. Western blot pictures are representative of at least three independent experiments. Asterisks indicate significant differences between experimental groups (*, *p* < 0.05, **, *p* < 0.01, ***, *p* < 0.001, ****, *p* < 0.0001).

## 3. Results

**Figure 1:**
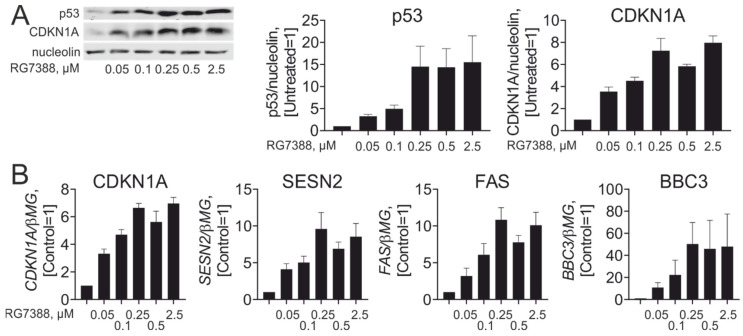
Idasanutlin concentration-dependently activates p53 in human macrophages. (**A**) Western analysis and quantification of p53 and CDKN1A (p21) protein expression in macrophages exposed to indicated concentrations of RG7388 for 24 h (*n* = 4). (**B**) mRNA expression of indicated p53 target genes in macrophages treated with different concentrations of RG7388 for 24 h (*n* = 3).

To study the effects of idasanutlin (RG7388) in primary human macrophages, we initially determined effective concentrations of idasanutlin towards p53 protein stabilization and p53-dependent transcriptional responses in M-CSF-differentiated human monocyte-derived macrophages. As shown in Figure 1A, idasanutlin potently increased cellular levels of p53 protein, starting at 50 nM and reaching a plateau at 250 nM, in accordance with findings in cancer cell lines [14,15]. A similar concentration-dependent effect was observed when mRNA and protein expression of classical p53 target genes, such as CDKN1A (p21), was analyzed (Figure 1A,B). Based on these data, we used 250 nM idasanutlin in all subsequent experiments.

**Figure 2:**
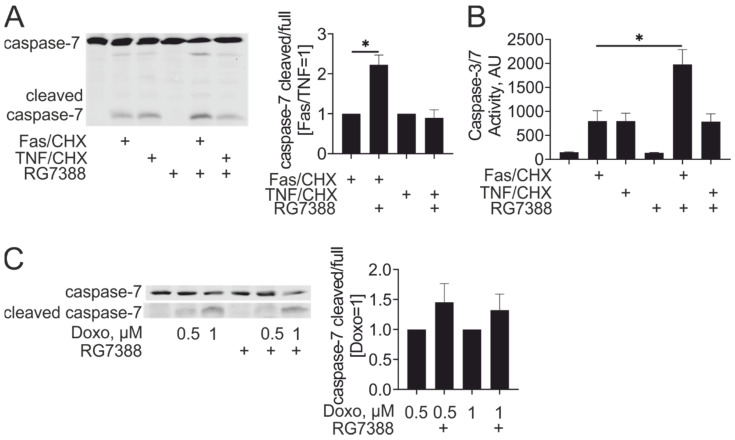
Idasanutlin sensitizes macrophages to apoptosis by the Fas-stimulating antibody. (**A**,**B**) Western analysis and quantification of caspase-7 cleavage (**A**) and caspase-3/7 activity (**B**) in macrophages pre-treated with 250 nM RG7388 for 24 h followed by treatments with 1 µg/mL Fas antibody or 10 ng/mL TNFα in the presence of 10 µg/mL cycloheximide (CHX) for 6 h (*n* = 4). (**C**) Western blot analysis and quantification of caspase-7 cleavage in macrophages pre-treated with 250 nM RG7388 for 24 h followed by treatments with doxorubicin for 24 h (*n* = 5).

p53 transcriptional targets include pro-apoptotic proteins, such as Puma or Fas. Their induction by idasanutlin was confirmed in our system (Figure 1B). To examine whether increased pro-apoptotic gene expression sensitized idasanutlin-treated macrophages to apoptosis, we pre-treated macrophages with idasanutlin, and exposed them to apoptosis-inducing Fas agonistic antibody or tumor necrosis factor α (TNFα) in the presence of cycloheximide. Apoptosis was analyzed following the cleavage of the effector caspase-7 by Western blotting. Idasanutlin increased caspase-7 cleavage in Fas antibody/cycloheximide-treated cells without affecting the sensitivity to TNFα/cycloheximide (Figure 2A). Similar results were obtained when analyzing caspase-3/7 enzymatic activity in lysates of macrophages stimulated with Fas antibody or TNFα in the presence of cycloheximide after idasanutlin pre-treatment (Figure 2B). We also assessed whether idasanutlin pre-treatment increased macrophage sensitivity to apoptosis after treatment with the DNA damage-inducing chemotherapeutic drug doxorubicin. In analyzing caspase-7 cleavage, we found that although there was a tendency towards increased caspase-7 cleavage after idasanutlin pre-treatment, it did not reach statistical significance (Figure 2C). Taken together, our findings indicate that pharmacological p53 activation specifically enhances apoptosis induced by Fas ligation without generally increasing apoptotic sensitivity of human macrophages.

**Figure 3:**
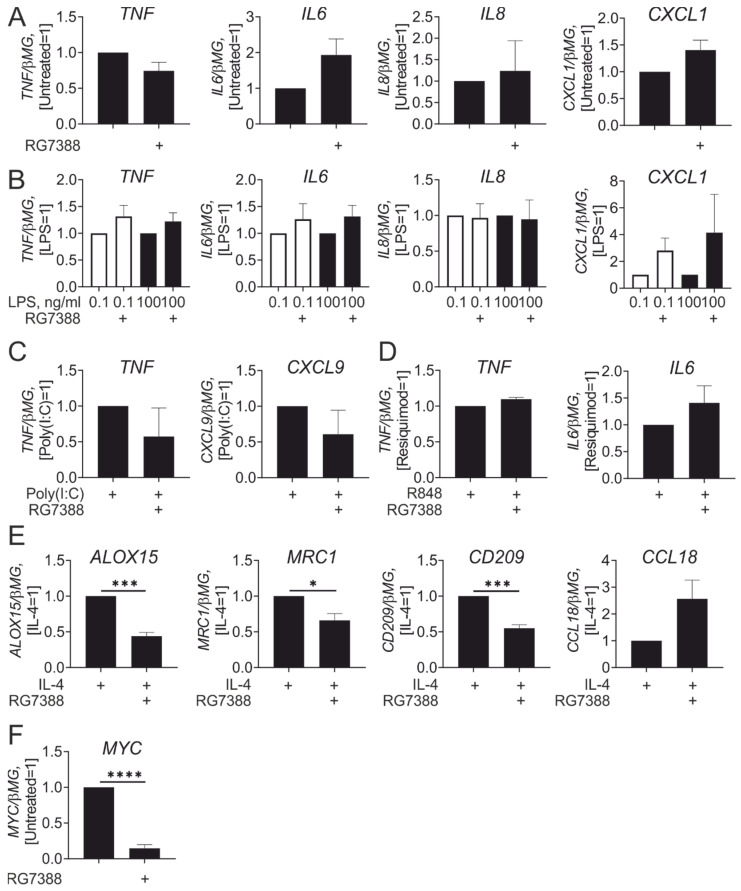
Idasanutlin inhibits IL-4-induced gene expression without affecting lipopolysaccharide (LPS) responses in human macrophages. (**A**,**B**) mRNA expression of indicated genes in macrophages treated with 250 nM RG7388 for 24 h (**A**) followed by treatments with 0.1 and 100 ng/mL LPS (**B**) for 3 h (*n* = 6). (C, D) mRNA expression of indicated genes in macrophages treated with 250 nM RG7388 for 24 h followed by treatments with 25 µg/mL poly (I:C) (**C**) or 1 µg/mL R848 (**D**) for 3 h (*n* = 3). (**E**) mRNA expression of indicated genes in macrophages treated with 250 nM RG7388 for 24 h followed by treatments with 20 ng/mL IL-4 for 24 h (*n* = 6). (F) MYC mRNA expression in macrophages treated with 250 nM RG7388 for 24 h (*n* = 6).

Next, we assessed inflammatory responses of idasanutlin-pre-treated macrophages. p53 protein stabilization by the MDM2 inhibitor Nutlin-3 was reported to induce pro-inflammatory human macrophage polarization through activation of NFκB [5]. However, pre-incubation with idasanutlin for 24 h did not significantly affect mRNA expression of pro-inflammatory cytokines, i.e., IL-6, TNFα, neutrophil chemokines IL-8, or chemokine (C-X-C motif) ligand 1 (CXCL1) (Figure 3A). In addition, idasanutlin failed to potentiate up-regulation of these genes in macrophages responding to bacterial lipopolysaccharide (LPS), either at low (100 pg/mL) or high (100 ng/mL) concentrations (Figure 3B). Idasanutlin pre-treatment also left pro-inflammatory responses towards stimulation of TLR3 with Poly(I:C) (Figure 3C) or TLR7/8 with R848 unaffected (Figure 3D). However, and in accordance with a previous observations in murine macrophages [16], idasanutlin attenuated IL-4-induced target gene expression (Figure 3E). Consistent with a suggested mechanism of p53 action [16], idasanutlin strongly suppressed Myc mRNA expression (Figure 3F). Indeed, Myc-dependent IL-4 target genes reported for human macrophages [17], such as ALOX15, CD209, or MRC1, were suppressed by idasanutlin, whereas Myc-independent CCL18 was not (Figure 3E). Together, our data do not support pro-inflammatory effects associated with p53 activation, while confirming the inhibitory effect on IL-4-induced polarization in mature human macrophages.

**Figure 4:**
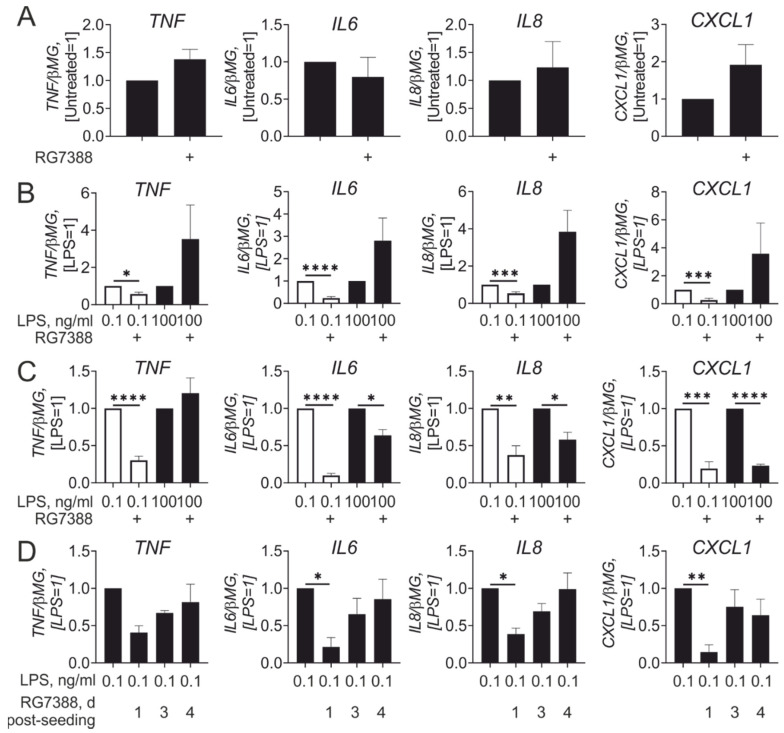
Idasanutlin added during monocyte to macrophage differentiation inhibits inflammatory responses to low-dose LPS. (**A**,**B**) mRNA expression of indicated genes in macrophages differentiated with M-CSF in the presence of 250 nM RG7388 for 7 days (**A**) followed by treatments with 0.1 and 100 ng/mL LPS (**B**) for 3 h (*n* = 4). (**C**) mRNA expression of indicated genes in macrophages differentiated with granulocyte-macrophage colony-stimulating factor (GM-CSF) in the presence of 250 nM RG7388 for 7 days followed by treatments with 0.1 and 100 ng/mL LPS for 3 h (*n* = 6). (**D**) mRNA expression of indicated genes in macrophages treated with 250 nM RG7388 at indicated times post-seeding during 7-day differentiation followed by treatments with 0.1 ng/mL LPS for 3 h (*n* = 4).

We then asked how p53 activation during monocyte to macrophage differentiation influences macrophage inflammatory responses. In these experiments, idasanutlin was added directly after monocyte isolation, and was present during the whole time course (7 days) of differentiation. This treatment resulted in sustained up-regulation of the p53 target CDKN1A, but did not induce apoptosis as assessed by analyses of caspase-7 cleavage and enzymatic caspase-3/7 activity (Figure S2). In analyzing basal expression of inflammatory cytokines, we did not notice significant differences between untreated and idasanutlin pre-treated macrophages (Figure 4A). However, macrophages differentiated in the presence of idasanutlin showed considerable differences in their response to LPS as compared with idasanutlin-treated mature macrophages. Whereas the response towards high LPS concentrations showed a tendency to increase without reaching statistical significance, the response to low LPS concentrations was significantly suppressed after idasanutlin treatment (Figure 4B). A similar inhibitory effect at low LPS concentrations was obtained when macrophages were differentiated in the presence of GM-CSF instead of M-CSF (Figure 4C). Time course experiments showed that idasanutlin should be added early during differentiation to exert an inhibitory effect on LPS-induced macrophage polarization (Figure 4D).

**Figure 5:**
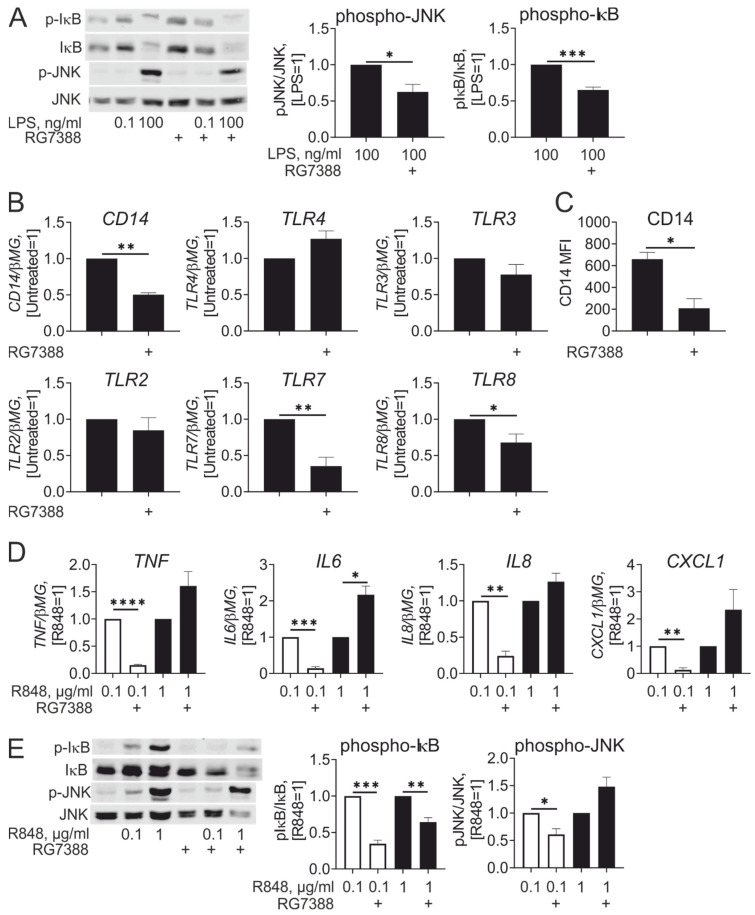
Idasanutlin inhibits expression of CD14, TLR7, and TLR8, attenuating inflammatory response to R848. (**A**) Western analysis and quantification of IκB and JNK phosphorylation in macrophages following differentiation in the presence of 250 nM RG7388 and treatments with 0.1 and 100 ng/mL LPS (*n* = 6). (**B**,**C**) mRNA expression of indicated genes (**B**, *n* = 5) and CD14 cell surface expression (**C**, *n* = 4) in macrophages differentiated with M-CSF in the presence of 250 nM RG7388 for 7 days. (**D**,**E**) mRNA expression of indicated genes (**D**) and Western blot analysis and quantification of IκB and JNK phosphorylation (**E**) in macrophages following differentiation in the presence of 250 nM RG7388 and treatments with 0.1 and 1 µg/mL R848 (*n* = 4).

Next, we addressed the possible causes of the inhibitory effect of idasanutlin on LPS-induced inflammatory responses. First, we analyzed LPS-induced intracellular signaling cascades. Examining phosphorylation of IκB and c-Jun N-terminal kinase (JNK), we found that idasanutlin added during differentiation attenuated activation of NFκB and JNK signaling after LPS stimulation even at high concentrations (Figure 5A). We reasoned that idasanutlin may then affect very early events of LPS-induced signaling. Analyzing mRNA expression of LPS receptors TLR4 and CD14, we noticed that TLR4 mRNA was not affected, while CD14 was significantly reduced in idasanutlin-treated cells (Figure 5B). Idasanutlin also reduced surface expression of CD14 (Figure 5C). Thus, attenuated responses of macrophages differentiated in the presence of idasanutlin to low LPS concentrations are associated with reduced expression of LPS co-receptor CD14.

Examining expression of other TLRs, we noticed that idasanutlin, when present during monocyte to macrophage differentiation, reduced mRNA expression of TLR7 and TLR8, while leaving mRNA levels of TLR2 and TLR3 unaffected (Figure 5B). Therefore, we tested whether reduced expression of TLR7/8 attenuates responses to stimulation with a TLR7/8 agonist. Indeed, macrophages differentiated in the presence of idasanutlin, displayed reduced levels of pro-inflammatory cytokine/chemokine mRNAs upon stimulation with low (0.1 µg/mL) concentrations of R848 (Figure 5D). Interestingly, at higher (1 µg/mL) concentration of R848, idasanutlin-treated macrophages displayed a tendency of enhanced inflammatory cytokine/chemokine mRNA expression. Only for IL-6 did this reach statistical significance. In analyzing activation of NFκB and JNK pathways by R848 by following IκB and JNK phosphorylation, we observed that whereas macrophages differentiated in the presence of idasanutlin showed reduced IκB phosphorylation in response to both 0.1 µg/mL and 1 µg/mL R848, attenuation of JNK phosphorylation was only observed at low dose of R848 (Figure 5E). Thus, idasanutlin reduces the sensitivity of human macrophages towards stimulation with low doses of TLR7/TLR8 agonists.

## 4. Discussion

The impact of p53 on inflammatory responses remains unresolved. Studies performed in p53 knockout mice and macrophages suggested an anti-inflammatory role of p53 [3,4,18]. However, further work using human macrophages indicated strong pro-inflammatory effect of pharmacological p53 activation with a first generation agent targeting the p53-MDM2 interaction, Nutlin-3 [5]. Further development of MDM2 antagonists with improved potency advanced these drugs to clinical trials [19]. However, their impact on the cells of the immune system remains largely unexplored. Understanding the role of p53 in modulating inflammatory responses is very important, considering the growing number of anti-cancer treatments aiming to activate the innate immune system. Thus, it is important to understand whether p53-activating pharmacological interventions have a positive or negative influence on anti-tumor immunity. To address this question using a translationally relevant system of human primary macrophages, we examined the effects of a new generation of MDM2 antagonist idasanutlin on macrophage inflammatory responses. Our data reveal differential roles of p53 activation in mature vs. differentiating macrophages. The prevailing effect is an anti-inflammatory action of p53 during differentiation due to the negative influence on the expression of several pattern recognition receptors.

Since several pro-apoptotic genes are prominent in the p53-dependent transcriptome, we initially investigated the impact of idasanutlin on macrophage sensitivity to a range of apoptosis-inducing agents. Interestingly, we did not observe a general increase of apoptosis after p53 activation, in spite of upregulation of known effectors of the intrinsic branch of pro-apoptotic signaling, such as BBC3 (Puma) [20]. p53 activation during monocyte to macrophage differentiation was well tolerated. This indicates that pharmacological p53 activation is generally unlikely to reduce macrophage populations through activation of pro-apoptotic signaling. However, in accordance with a strong up-regulation of Fas mRNA, we observed potentiation of a pro-apoptotic response to a Fas-stimulating antibody. This provides a possibility to eliminate macrophages by combining Fas- and p53-activating pharmacological interventions.

In contrast to previous findings in Nutlin-3-treated macrophages [5], we did not observe increased expression of pro-inflammatory cytokines after p53 activation by idasanutlin. Idasanutlin also failed to potentiate inflammatory effects of TLR4, TLR3, or TLR7/8 agonists. Our data argue that p53 activation in fully differentiated macrophages is neutral with regard to their inflammatory potential. While we noticed some difference in the experimental setup between our study and that of Lowe et al., e.g., using 24 h treatments with idasanutlin vs. 2–6 h stimulations with Nutlin-3, idasanutlin failed to induce pro-inflammatory gene expression also after shorter incubation times (data not shown). Therefore, Nutlin-3 may elicit pro-inflammatory macrophage activation through p53-independent mechanisms.

On the other hand, idasanutlin suppressed IL-4-induced gene expression in accordance with previous findings in murine macrophages [16]. Since we observed a strong down-regulation of Myc mRNA expression after idasanutlin treatment, and Myc is known to support IL-4-triggered polarization of human macrophages [17], p53 activation likely attenuates transcriptional response to IL-4 by targeting Myc [21]. This is also supported by the observation that up-regulation of a Myc-independent target gene CCL18 in response to IL-4 was not affected by idasanutlin. As IL-4-induced macrophage polarization is considered anti-inflammatory and may be relevant for shaping the immunosuppressive phenotype of tumor-associated macrophages [22,23], this effect of p53 activation may support anti-tumor immunity.

In contrast to relative inertness of idasanutlin with respect to inflammatory properties of mature macrophages, idasanutlin added during monocyte to macrophage differentiation suppressed inflammatory responses to TLR4 and TLR7/8 agonists. This correlated with reduced expression of TLR7 and TLR8 as well as attenuated expression of the TLR4 co-receptor CD14. Our experiments did not directly address causality between their expression and attenuated inflammatory responses of idasanutlin pre-treated macrophages. However, this mechanism is supported by observations that major branches of TLR-induced signaling were inhibited by idasanutlin, suggesting a defect early in the cascade. In addition, the inhibitory effect of idasanutlin was observed only at low concentrations of TLR4 and TLR7/8 agonists, suggesting that idasanutlin causes a rightward shift of the TLR ligand concentration-dependence curve, typical for a situation when the amount of receptor is reduced. Our data are in contrast to observations in T lymphocytes, where p53 activation induced up-regulation of several TLRs [24], including TLR3 and TLR4. However, in the same publication, human alveolar macrophages did not respond to p53 activation. Therefore, the impact of p53 on TLR expression appears to be cell type-specific [25].

At high concentrations of TLR agonists, we noticed that idasanutlin tended to increase pro-inflammatory mRNA expression, although the effects were highly variable and did not reach statistical significance. Idasanutlin moderately increased (2-fold and 1.5-fold, data not shown) the transcription factor IRF5, a known p53 target gene [26] with pro-inflammatory properties [27,28], which could underlie small pro-inflammatory effects of p53.

It is still unclear why p53 affects TLR expression during differentiation while having no effect on mature macrophages. p53 should be activated early during differentiation to exert anti-inflammatory effects. Whether this requires direct genomic action of p53 or is mediated by some p53 target genes remains to be elucidated. It should be noted that p53 does not generally interfere with the macrophage differentiation process, since expression of different phagocytic receptors and differentiation markers (CD11b, CD64, CD16, CD40) remained intact in the presence of idasanutlin (data not shown).

## 5. Conclusions

The results of our study show that pharmacological p53 activation during monocyte to macrophage differentiation attenuates macrophage responsiveness to immunostimulatory TLR agonists. Regarding the implications for immunotherapy, our data suggest that p53 activation may not be effective at enhancing anti-tumor properties of macrophages. Furthermore, by interfering with TLR7/8, p53 activation may be detrimental for anti-cancer immunotherapies targeting these receptors [29,30]. This should be taken into account in future designs of combination chemo- and immunotherapies.

## Figures and Tables

**Table 1:** List of the primers used in Q-PCR analyses.

Gene	Forward	Reverse
β-microglobulin	TCCAAAGATTCAGGTTTACTCA	ATATTAAAAAGCAAGCAAGCAG
CDKN1A	CCCAGTTCATTGCACTTTGATTAGC	ACAGTCTAGGTGGAGAAACGGGAAC
SESN2	AAGGACTACCTGCGGTTCG	CGCCCAGAGGACATCAGTG
FAS	GTGACCCTTGCACCAAATGT	AGACAAAGCCACCCCAAGTT
BBC3	AGAGCAGGGCAGGAAGTAAC	GAGGGCTGAGGACCACAAAT
TNF	GACAAGCCTGTAGCCCATGT	GAGGTACAGGCCCTCTGATG
IL6	TCCACAAGCGCCTTCGGTCC	TCAGGGCTGAGATGCCGTCG
IL8	AGCCTTCCTGATTTCTGCAGCTCT	AATTTCTGTGTTGGCGCAGTGTGG
CXCL1	TCGCCAGCTCTTCCGCTC	CACGGACGCTCCTGCTG
CXCL9	CCACCGAGATCCTTATCGAA	GCTAACTGGGCACCAATCAT
ALOX15	TGGAAGGACGGGTTAATTCTGA	GCGAAACCTCAAAGTCAACTCT
MRC1	GGCGGTGACCTCACAAGTAT	ACGAAGCCATTTGGTAAACG
CD209	AGCTGTGGCCCCCAGGAGTT	ACCATGGCCAAGACACCCTGC
CCL18	CCCAGCTCACTCTGACCACT	GTGGAATCTGCCAGGAGGTA
MYC	TTCGGGTAGTGGAAAACCAG	CAGCAGCTCGAATTTCTTCC
CD14	GACCTAAAGATAACCGGCACC	GCAATGCTCAGTACCTTGAGG
TLR4	AAAATCCCCGACAACCTCCC	TGTCTGGATTTCACACCTGGA
TLR3	GCTAGCAGTCATCCAACAGAATC	TGGCGGCTGGTAATCTTCTG
TLR2	GGGTCATCATCAGCCTCTCC	AGGTCACTGTTGCTAATGTAGGTG
TLR7	GGCCCATCTCAAGCTGATCT	GTGTCCACATTGGAAACACCATT
TLR8	TGGGAAAGGAGACTAAAAAGGAAA	TCTTCGGCGCATAACTCACA

## Data Availability

The primary data generated in this study are available upon request from the corresponding author.

## Supplementary Materials

The following are available online at https://www.mdpi.com/2072-6694/13/5/958/s1 Figure S1: Uncropped images of Western Blots, Figure S2: Caspase-3/7 activity in macrophages differentiated with M-CSF in the presence of 250 nM RG7388 for 6 days.
