# Supplementary material for: Pharmacological Activation of p53 during Human Monocyte to Macrophage Differentiation Attenuates Their Pro-Inflammatory Activation by TLR4, TLR7 and TLR8 Agonists
Source: Cancers (Basel). 2021 Feb 25;13(5):958. doi: 10.3390/cancers13050958 (PMC7956237; doi:10.3390/cancers13050958)
Supplement: Supplementary file 1 [file cancers-13-00958-s001.pdf]

cancers

MDPI

Supplementary Materials: Pharmacological Activation of p53 during Human Monocyte to Macrophage Differentiation Attenuates Their Pro-Inflammatory Activation by TLR4, TLR7 and TLR8 Agonists.

Dmitry Namgaladze and Bernhard Brüne

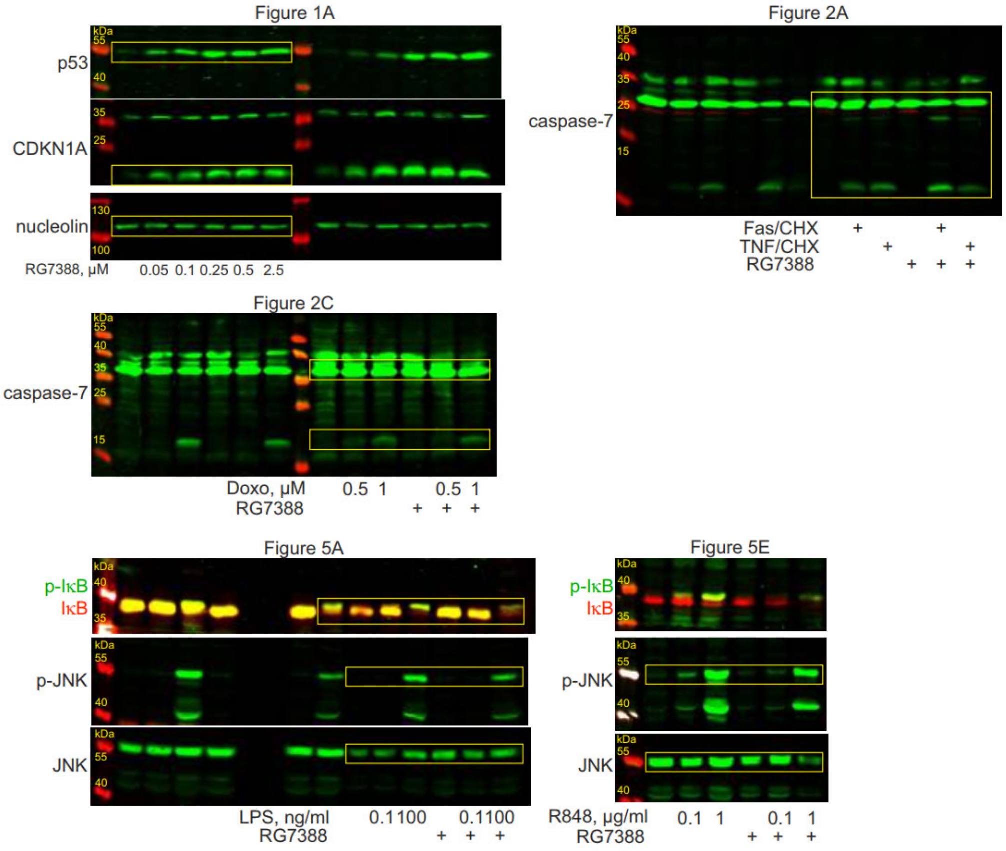

Figure S1. Uncropped images of Western Blots.

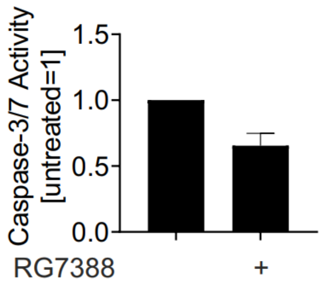

Figure S2. Caspase-3/7 activity in macrophages differentiated with M-CSF in the presence of 250 nM RG7388 for 6 days.
